# Ectopic hyperparathyroidism due to an auto‐transplanted parathyroid gland

**DOI:** 10.1002/ccr3.3136

**Published:** 2020-07-16

**Authors:** Ko Harada, Fumio Otsuka

**Affiliations:** ^1^ Department of General Medicine Okayama University Graduate School of Medicine, Dentistry, and Pharmaceutical Sciences Okayama Japan

**Keywords:** auto‐transplantation, ectopic hyperparathyroidism, hypercalcemia, primary hyperparathyroidism

## Abstract

Hyperparathyroidism due to the autografted parathyroid gland should be suspected in patients who undergone parathyroid auto‐transplantation.

## Graphical abstract

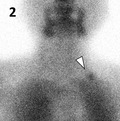

## 1 CASE PRESENTATION

Although parathyroid tissue auto‐transplantation is useful for preventing hypoparathyroidism during thyroidectomy, hyperparathyroidism may occur due to proliferation of the transplanted parathyroid tissue. Hyperparathyroidism due to the autografted parathyroid gland should be suspected in patients who undergone parathyroid auto‐transplantation.

**FIGURE 1:**
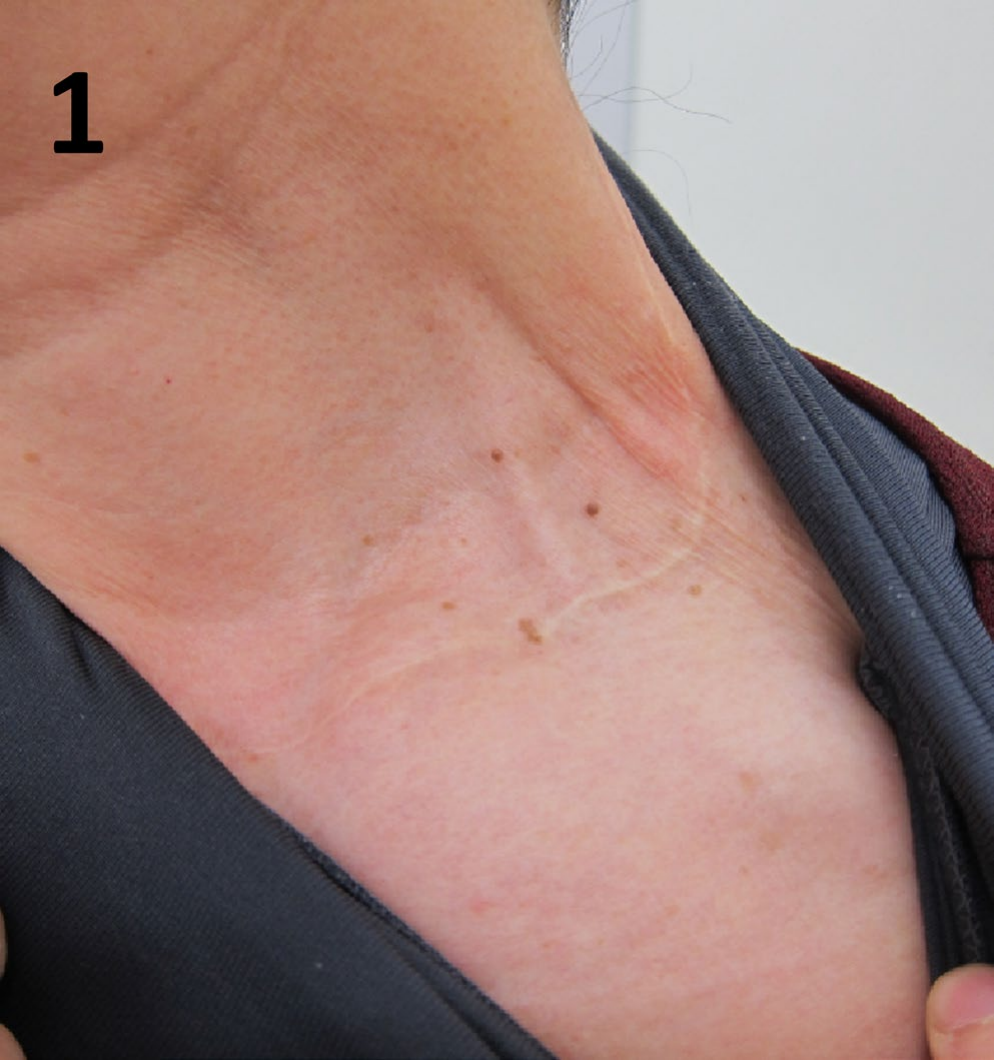
On physical examination, a surgical scar on the left side of her neck was observed

**FIGURE 2:**
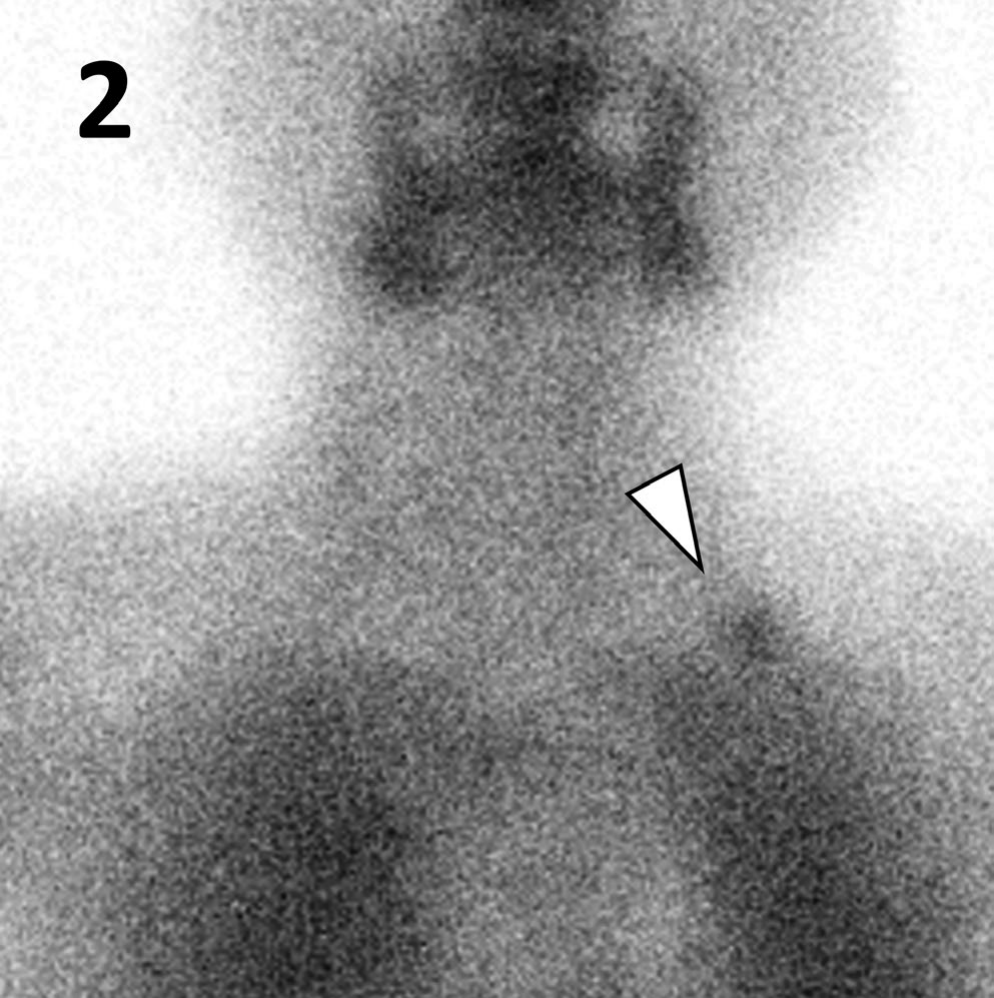
Tc‐99m‐MIBI demonstrated increased uptake in the autograft in the left supraclavicular region (arrowhead)

A 65‐year‐old woman was referred to our hospital because of hypercalcemia. She had undergone total thyroidectomy for thyroid cancer 30 years prior along with left‐sided parathyroid auto‐transplantation in her neck, without any family history of endocrine diseases. Physical examination showed a surgical scar on the left side of her neck (Figure 1). Her serum calcium, phosphate, and intact parathyroid hormone levels were 11.3 mg/dL, 3.4 mg/dL, and 170 pg/mL, respectively. Tc‐99m‐MIBI demonstrated increased uptake in the autograft in the left supraclavicular region (Figure 2). Based on these findings, primary hyperparathyroidism due to the autografted parathyroid gland was strongly suspected.

Although parathyroid tissue auto‐transplantation is useful for preventing hypoparathyroidism during thyroidectomy, hyperparathyroidism may occur due to proliferation of the transplanted parathyroid tissue.^1^, ^2^ The reported recurrence rate of secondary hyperparathyroidism after auto‐transplantation of hyperplastic parathyroid tissue is 3%‐14%.^3^ For asymptomatic patients with ectopic hyperparathyroidism, surgical intervention is recommended if serum calcium concentration is 1.0 mg/dL or more above the normal upper limit.^4^ As our patient was reluctant to undergo surgery, surgery was not performed, and serum calcium levels were monitored. Our case suggests that hyperparathyroidism due to autografted parathyroid should be suspected in patients who undergo parathyroid auto‐transplantation.

## CONFLICT OF INTERESTS

None declared.

## AUTHOR CONTRIBUTION

KH: contributed to patient care, writing the manuscript, and discussion. FO: revised the manuscript.

## ETHICAL APPROVAL

Written consent was obtained from the patient.
